# SomInaClust: detection of cancer genes based on somatic mutation patterns of inactivation and clustering

**DOI:** 10.1186/s12859-015-0555-7

**Published:** 2015-04-23

**Authors:** Jimmy Van den Eynden, Ana Carolina Fierro, Lieven PC Verbeke, Kathleen Marchal

**Affiliations:** 1 Department of Information Technology, Ghent University - iMinds, Ghent, Belgium; 2 Department of Plant Biotechnology and Bioinformatics, Ghent University, Ghent, Belgium

**Keywords:** Cancer, Mutation, Driver gene, Oncogene, Tumour suppressor gene

## Abstract

**Background:**

With the advances in high throughput technologies, increasing amounts of cancer somatic mutation data are being generated and made available. Only a small number of (driver) mutations occur in driver genes and are responsible for carcinogenesis, while the majority of (passenger) mutations do not influence tumour biology. In this study, SomInaClust is introduced, a method that accurately identifies driver genes based on their mutation pattern across tumour samples and then classifies them into oncogenes or tumour suppressor genes respectively.

**Results:**

SomInaClust starts from the observation that oncogenes mainly contain mutations that, due to positive selection, cluster at similar positions in a gene across patient samples, whereas tumour suppressor genes contain a high number of protein-truncating mutations throughout the entire gene length. The method was shown to prioritize driver genes in 9 different solid cancers. Furthermore it was found to be complementary to existing similar-purpose methods with the additional advantages that it has a higher sensitivity, also for rare mutations (occurring in less than 1% of all samples), and it accurately classifies candidate driver genes in putative oncogenes and tumour suppressor genes. Pathway enrichment analysis showed that the identified genes belong to known cancer signalling pathways, and that the distinction between oncogenes and tumour suppressor genes is biologically relevant.

**Conclusions:**

SomInaClust was shown to detect candidate driver genes based on somatic mutation patterns of inactivation and clustering and to distinguish oncogenes from tumour suppressor genes. The method could be used for the identification of new cancer genes or to filter mutation data for further data-integration purposes.

**Electronic supplementary material:**

The online version of this article (doi:10.1186/s12859-015-0555-7) contains supplementary material, which is available to authorized users.

## Background

Carcinogenesis is a multi-step process caused by the accumulation of somatic mutations (point mutations or genomic copy number variations). These so-called driver mutations lead to a selective growth advantage of affected cells, resulting in positive selection and clonal expansion [1-3]. Driver mutations occur in cancer (driver) genes, which are involved in cell proliferation, differentiation and apoptosis. A distinction is made between oncogenes (OGs) and tumour suppressor genes (TSGs). OGs lead to a growth advantage when they become constitutively active due to gain-of-function mutations. For TSGs this growth advantage is explained by inactivation of the gene as a result of loss-of-function or protein-truncating mutations (nonsense mutations and frameshift insertions or deletions) [4,5].

However, driver mutations only represent a minority of the total number of mutations that are present in a malignant tumour [3]. Indeed, most mutations that are found in (cancer) cells are random events that do not lead to growth advantages. These mutations are known as passenger mutations. While it is estimated that the development of a malignant tumour implies between 5 to 8 driver mutations, a median number of 33 to 66 mutations are found in most solid tumours, from which the majority are passenger mutations [1]. With the increasing number of whole genome and exome data made available via initiatives like TCGA and ICGC [6,7], distinguishing driver mutations from passenger mutations has become challenging. This distinction is not only important for the identification of novel cancer genes as such, but also because a lot of the currently available data-integration methods depend on a reliable filtering of somatic mutations.

This filtering is often frequency-based, i.e. the analysis is restricted to genes that are mutated in a minimal proportion of all samples (e.g. 2%) [8-10]. However, this approach implies that non-frequently mutated genes will be discarded and crucial information might get lost for further analysis. On the other hand it is well-known that some genes that are not involved in carcinogenesis (e.g. TTN) might be frequently mutated and hence will be selected by a naïve frequency-based filtering method [11]. Therefore, several driver gene prioritization methods have been developed recently that search for signs of positive selection across a cohort of tumours to identify candidate driver genes [12]. Methods like MutSig(CV) [11], MuSiC [13] and OncodriveClust [14] detect genes that are mutated more frequently than expected from a calculated background mutation rate, while methods like OncodriveFM [15] and ActiveDriver [16] assess the expected functional impact to identify putative driver genes. However, these approaches are in general not capable of detecting less frequently mutated genes, nor do they distinguish between OGs and TSGs.

Vogelstein et al. suggested a ratiometric method (or “20/20 rule”) to detect driver genes and distinguish between OGs and TSGs [1]. This method is based on the assumption that in OGs at least 20% of the mutations are missense mutations that cluster at recurrent positions across different tumour samples, whereas for TSGs at least 20% of the observed mutations are protein-truncating mutations which occur throughout the entire gene length. To be classified as an oncogene, an arbitrary threshold of 10 clustering mutations was required and the classification of tumour suppressor genes implied a minimal number of 7 inactivating mutations. Furthermore the scores assigned to the different genes cannot be interpreted statistically and the method requires additional manual curation for its application [1].

To solve these issues we have developed SomInaClust, which builds on the basic concept of Vogelstein’s method. The method uses a large reference mutation database to 1) determine mutational hot spots, i.e. CDS (coding DNA sequence) positions that contain more mutations across samples than could be expected purely by chance and 2) calculate the gene-specific background mutation rate. This information is subsequently used to determine candidate driver genes in a specific set of mutation data, based on the mutation pattern that is observed in each gene across tumour samples. SomInaClust was able to accurately prioritize driver genes in 9 different solid cancers and allowed distinguishing OGs from TSGs in these tumours, even for genes that have a low mutation frequency.

## Methods

### Overview of SomInaClust

SomInaClust identifies candidate driver genes from whole exome or genome mutation data based on their mutation patterns. The basic assumption is that, due to selective pressure, driver genes are characterized by 1) clustering mutations and/or 2) a high number of inactivating (protein-truncating) mutations across tumour samples. Whereas the former is the main pattern expected for OGs, the latter is the main pattern for TSGs. Because of this association, inactivating mutations (i.e. nonsense mutations and frameshift indels) are further referred to as TSG mutations and other mutations (i.e. missense mutations and in-frame indels) are referred to as OG mutations. The method follows a two-step approach (Additional file 1: Figure S1).

In the **first or reference step** the background mutation rate is estimated for each gene and hot spots (i.e. gene positions where mutations tend to cluster across tumour samples) are identified in a reference mutation database (Additional file 1: Figure S1). In this study the COSMIC database was used for this purpose [17].

Mutation rates are known to vary widely throughput the genome [18]. To correct for this mutational heterogeneity, two gene-specific correction factors are calculated. This calculation starts from the simplifying assumption that mutations have an equal occurrence probability and that silent mutations are not under selective pressure. Starting from 61 different non-stop codons, each with 9 possible mutations (i.e. 3 for each nucleotide), 549 different mutations can theoretically occur. From those 549 mutations, 392 can be classified as missense, 23 as nonsense and 134 as silent mutations (Additional file 2: Table S1). Assuming an equal mutation probability, a ratio of 134/392 silent-to-missense mutations (i.e. for each 392 missense mutations, 134 silent mutations are to be expected) and 134/23 silent-to-nonsense mutations would be expected. By comparing for each gene the observed ratios to these expected ratios, two gene-specific correction factors are defined:

$$ \begin{array}{ccc}\hfill -\hfill & \hfill \mathrm{O}\mathrm{G}\ \mathrm{correction}\ \mathrm{factor}:\hfill & \hfill 1-\frac{observed\  ratio}{expected\  ratio}=1-\frac{\raisebox{1ex}{${N}_{sil}$}\!\left/ \!\raisebox{-1ex}{${N}_{OG}$}\right.}{\raisebox{1ex}{$134$}\!\left/ \!\raisebox{-1ex}{$392$}\right.}\hfill \end{array} $$

$$ \begin{array}{ccc}\hfill -\hfill & \hfill \mathrm{T}\mathrm{S}\mathrm{G}\ \mathrm{correction}\ \mathrm{factor}:\hfill & \hfill 1-\frac{observed\  ratio}{expected\  ratio}=1-\frac{\raisebox{1ex}{${N}_{sil}$}\!\left/ \!\raisebox{-1ex}{${N}_{TSG}$}\right.}{\raisebox{1ex}{$134$}\!\left/ \!\raisebox{-1ex}{$23$}\right.}\hfill \end{array} $$

with N_sil_, N_OG_ and N_TSG_ being the total number of observed silent mutations, OG mutations and TSG mutations in a gene across tumour samples. When the correction factors are negative (observed ratio > expected ratio), they are put to zero. By now multiplying, for each analysed gene the observed number of OG or TSG mutations with these gene-specific correction factors, mutations are corrected for different background mutation rates. Genes that contain a high number of mutations due to a high background mutation rate will have a correction factor close to zero (number of silent mutations will be proportionally increased). In these genes OG or TSG mutations are expected to be rather non-specific. This low correction factor will decrease the weight of these mutations. Genes that contain a high number of mutations due to positive selection will have a correction factor close to one, meaning a higher weight of the mutations.

Subsequently, for each gene the mutational hot spots are determined. A hot spot corresponds to any CDS nucleotide position for which at least k OG mutations are observed across samples and for which the probability of observing this same number of k OG mutations by chance is less than 0.05, given a total number of n OG mutations and the gene’s CDS length. This can be calculated using a cumulative binomial test given by:

$$ {\displaystyle \sum_1^{k_{OG}}}\left(\begin{array}{c}\hfill {n}_{OG}\hfill \\ {}\hfill {k}_{OG}\hfill \end{array}\right){p}_l^{k_{OG}-1}{\left(1-{p}_l\right)}^{n_{OG}-{k}_{OG}}\le 0.05 $$

Where p_l_ = 1/CDS length, k_OG_ = the number of clustering OG mutations on the same nucleotide position and n_OG_ = the total number of observed OG mutations in the gene across tumour samples (the latter two after correction with the OG correction factor).

In the **second or detection step** the gene-specific OG (pOG) and TSG (pTSG) random mutation probabilities are determined (Additional file 1: Figure S1).

pOG is defined as the probability that k_h_ random OG mutations occur at hot spot locations (defined in the first step), given a total number of n observed OG mutations for a given CDS length. This can be calculated using the following cumulative binomial test:

$$ pOG={\displaystyle \sum_1^{k_{hOG}}}\left(\begin{array}{c}\hfill {n}_{OG}\hfill \\ {}\hfill {k}_{hOG}\hfill \end{array}\right){p}_h^{k_{hOG}}{\left(1-{p}_h\right)}^{n_{OG}-{k}_{hOG}} $$

Where p_h_ = the probability for a random mutation to occur in a hotspot, i.e. the number of hot spots/CDS length. k_hOG_ = the number of OG mutations located at hot spots and n_OG_ = the total number of OG mutations across tumour samples (both after correction with the OG correction factor).

The same binomial test is used for the calculation of pTSG, but instead of using the probability of a random mutation to occur in a hotspot, the protein-truncating mutation probability is used, as estimated from the nonsense mutation probability:

$$ pTSG={\displaystyle \sum_1^{k_{TSG}}}\left(\begin{array}{c}\hfill {n}_{tot}\hfill \\ {}\hfill {k}_{TSG}\hfill \end{array}\right)\ {p}_n^{k_{TSG}}{\left(1-{p}_n\right)}^{n_{tot}-{k}_{TSG}} $$

Where p_n_ = the probability for a random mutation to be a nonsense mutation, here defined as 23/549 (23 ways for a non-stop codon to mutate in a stop codon out of 549 theoretically possible mutations, see Additional file 2: Table S1). k_TSG_ = the number of TSG mutations and n_tot_ = the total number of mutations across tumour samples (both corrected using the TSG correction factor).

Next, a multiple testing correction of pOG and pTSG using the Benjamini-Hochberg method is performed [19]. From the corrected p values (qOG and qTSG), the driver gene probability is calculated as follows: qDG = qOG x qTSG. A putative driver gene is then defined as a gene with qDG ≤ 0.05.

To classify predicted driver genes in respectively putative OGs or TSGs, two additional scores are defined: the OG score and the TSG score. The OG score is the proportion of (clustering) OG mutations to the total number of OG mutations. Putative OGs are then defined as genes with an OG score ≥20. In the same line the TSG score is the proportion of TSG mutations to the total number of mutations. Putative TSGs are then defined as genes with a TSG score ≥20. When both the OG and the TSG scores are ≥20, a gene is defined as a TSG (a specific missense mutation can render a TSG inactive, but a truncating mutation is rarely expected to overactivate a gene).

### Comparison with other driver gene identification algorithms

SomInaClust was compared with 3 alternative driver gene identification methods that could be run starting from the same maf (mutation annotation format) input file as SomInaClust: MutSigCV 1.4 [11], OncodriveClust [14] and OncodriveFM [15]. All algorithms were run in default mode, following the instructions provided by the developer.

**MutSigCV** was downloaded from http://www.broadinstitute.org/cancer/cga/mutsig and run following the instructions for the case where the analysis is based on the maf file only. MutSigCV was run in Matlab R2013a.

**OncodriveClust** was downloaded from https://bitbucket.org/bbglab/oncodriveclust/. The input files containing the protein affecting and coding silent mutations (i.e. gene names and amino acid positions) were generated from the maf files that were used by all other algorithms in this study. The input file containing the CDS length for the gene transcripts was created after downloading the HGNC (HUGO Gene Nomenclature Committee) gene name, Ensembl transcript ID and CDS length information from Biomart Ensembl.

**OncodriveFM** was downloaded from https://bitbucket.org/bbglab/oncodrivefm. OncodriveFM requires 1 input file containing, for each gene, 3 different functional impact scores: Mutation Assessor (MA), SIFT and PolyPhen (PP2) score. These scores were calculated using **Annovar** [20], which was downloaded from http://www.openbioinformatics.org/annovar/. Annovar input files were prepared using the maf file that was used by all other algorithms in this study. Missing MA, SIFT and PP2 were optimized using the criteria of A Gonzalez-Perez and N Lopez-Bigas [15]: 2,1 and 0 for silent mutations and 3.5,0 and 1 for nonsense or frameshift mutations.

### COSMIC and TCGA data

Somatic mutation reference data were downloaded from the **COSMIC database** on November 24, 2014 (v71) [21].

For the tumour-specific analysis, **TCGA whole exome data** were used [6]. The maf files were downloaded from the TCGA data portal on August 22, 2014. Only mutation data, analysed using the Illumina GA DNASeq platform and annotated using NCBI 37 (hg 19) were used for analysis. Data from the following cancer types were downloaded: bladder cancer (BLCA), breast cancer (BRCA), colon cancer (COAD), glioblastoma multiforme (GBM), head and neck squamous cell cancer (HNSC), lung squamous cell cancer (LUSC), ovarian cancer (OV), rectal cancer (READ) and uterine cancer (UCEC).

### Cancer gene census (CGC) list

The cancer census gene list (v71) was downloaded from COSMIC as a reference list for generally accepted cancer genes. This list is an ongoing effort to collect genes that contain mutations that have been shown to be involved in carcinogenesis [22]. It currently contains over 500 genes, including information on the molecular genetics of the gene, i.e. whether the gene operates in a dominant or in a recessive way. As OGs are expected to be dominant (i.e. one gain-of-function mutation allelic variant suffices for a growth advantage) and TSGs are expected to be recessive (i.e. one loss-of-function can still be compensated by the other allele) [4], this information was used to estimate the OG/TSG classification accuracy of the SomInaClust method.

### Pathway enrichment analysis using KEGG data

Pathway enrichment analysis was performed to determine the signalling pathways in which putative driver genes are active. Pathway interaction data were downloaded from KEGG (Kyoto Encyclopedia of Genes and Genomes [23]) on August 27, 2014 and pathway enrichment was determined by Fisher’s Exact Test with control of the false discovery rate (q ≤ 0.05).

### Data preprocessing and statistical analysis

The R statistical package was used for all data processing and statistical analysis, including the implementation of the SomInaClust method.

All gene names/symbols from all files used in this study (e.g. Cosmic, TCGA, CGC genes) were first converted to the currently approved HGNC gene symbols using the gene nomenclature of HGNC, downloaded on August 26, 2014. Because of small differences in the column names of the TCGA maf files (related to the centre that created the file), they were first converted to a uniform format as specified in the breast cancer (2.5.3) file. Only primary tumour samples were selected from these files.

When results were compared, they were first checked for normality using the Shapiro-Wilk test. In case of normal distribution the student t tests or ANOVA was used for data comparison and mean values were reported, otherwise the Wilcoxon-signed rank test was used and median values were reported. False discovery rate correction was always performed using the Benjamini-Hochberg method [19].

## Results

### Detection of mutational hot spots in the COSMIC database

Because it is difficult to detect clustering mutations and hence mutational hot spots across tumour samples in small-sized datasets, SomInaClust uses prior information regarding these gene-specific hot spots. This information is calculated from a large reference database.

**Figure 1:**
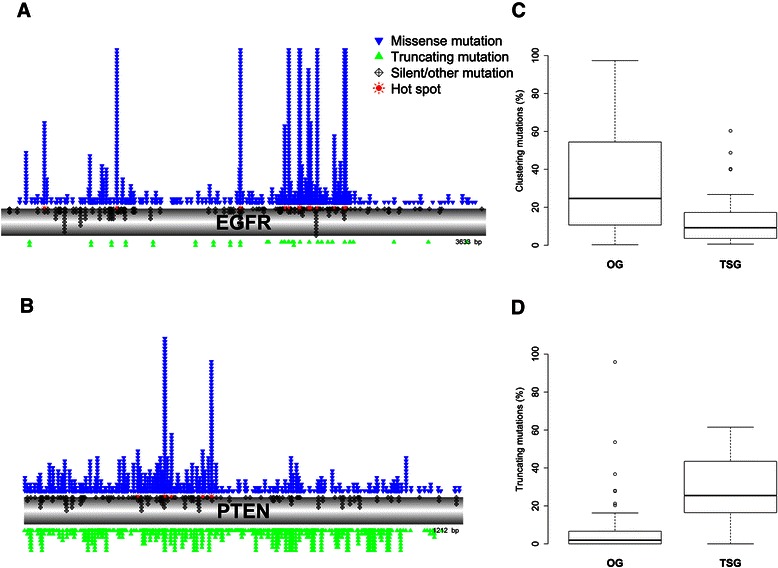
Observed mutation patterns for known cancer genes in the COSMIC reference database. Visualization of missense (blue), protein-truncating (green) and silent mutations (black) on the EGFR gene **(A)** as an example of a typical oncogene and the PTEN gene **(B)** as an example of a typical tumour suppressor gene. Hot spots (red) indicate gene positions where a significant clustering of mutations was detected by SomInaClust across tumour samples. **(C)** and **(D)**. Boxplots show the proportion of clustering **(C)** and protein-truncating **(D)** mutations for known oncogenes (OG) and tumour suppressor genes (TSG) for which hot spots were detected.

In this study we used COSMIC (v71) as a reference. This database is the most complete database of somatic mutations in cancer currently available [17], containing data from 257,740 samples with a total number of 2,726,304 mutations, occurring in 20,269 genes. A total number of 1,885 hot spots were detected in 867 genes, with a median number of 1 and a maximum number of 113 hot spots per gene (Additional file 3: Table S2). Mutation clusters (hot spots) were identified in both OGs (e.g. PIK3CA, EGFR, AKT1) and TSGs (e.g. TP53, PTEN, CDKN2A) (Figure 1). The identified mutational hot spots included many well-studied oncogenic mutations such as the PIK3CA’s E542K mutation (CDS position 1624 and 1625), the AKT’s E17K mutation (CDS position 49), and the EGFR’s L858R mutation (CDS position 2573) (Additional file 3: Table S2).

When the genes that are present in the CGC list (v71) were used as benchmark evaluation, 75% of CGC genes with detected clusters were OGs, while 25% were TSGs. Although these numbers and examples indicate that, as expected, for both OGs and TSGs significant clusters could be detected, the mutation pattern for both types was clearly different. For the known OGs, 25% (median) of all mutations were clustering mutations, while for the known TSGs, this was only 9% (p < 0.001). The opposite pattern was observed for protein-truncating mutations (2% vs. 25% of all mutations for OGs and TSGs respectively (p < 0.001)) (Figure 1).

These results confirm the different mutation patterns between OGs and TSGs across samples: OGs mainly contain clustering missense mutations, while TSGs also contain a high number of protein-truncating mutations.

### Identification of driver genes in TCGA cancer data

As the breast cancer dataset is one of the most extended and best characterized TCGA datasets [9], this study mainly focuses on breast cancer. The results from 8 other solid cancers are discussed in less detail and given in supplementary tables and figures.

**Figure 2:**
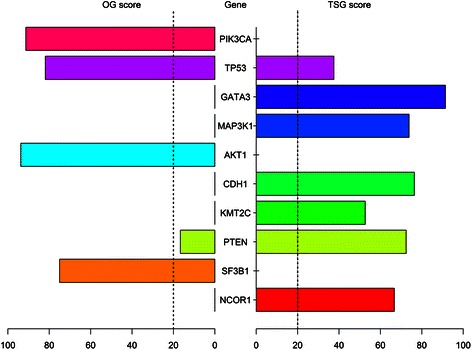
Top 10 putative driver genes identified in breast cancer. Pyramid plot showing the top 10 putative driver genes retrieved by SomInaClust on the TCGA breast cancer dataset and ranked according to their increasing q values. The OG scores are visualized on the left and the TSG scores on the right. Dotted lines indicate cut-offs for classification as oncogene or tumour suppressor gene.

Using the somatic mutation TCGA data from 772 primary breast tumours, containing 47,114 mutations occurring in 15,767 genes, SomInaClust identified 51 (0.3%) putative driver genes (Additional file 4: Table S3). Some of these genes were detected based on their high number of mutations located at mutational hot spots and hence high OG mutation contribution to the qDG value (e.g. PIK3CA, AKT1). Others were detected based on their high number of protein-truncating mutations and hence high TSG mutation contribution to the qDG value (e.g. GATA3, MAP3K1). For a third group of genes, both types of mutations contributed to their significant qDG values (e.g. TP53) (Figure 2). These results confirm the complementarity of using both OG and TSG mutation patterns in the identification of putative driver genes. Indeed, if only the qOG or qTSG values would be used for driver gene identification (based on the proportion of clustering mutations or protein-truncating mutations respectively) several cancer genes would have remained undetected (Additional file 5: Figure S2).

Some of the identified genes (e.g. TP53, PIK3CA, GATA3) are known to be frequently mutated driver genes in breast cancer [9]. However, SomInaClust also prioritized driver genes that are less frequently mutated in breast cancer like AKT1 (20 out of 772 mutations) and CDKN1B (7 mutations, representing less than 1% of all mutations across samples) (Additional file 6: Figure S3A). The opposite is true for other genes, like TTN, MUC4 and RYR2, which are amongst the most frequently mutated genes in breast cancer, but were clearly deprioritized by SomInaClust (Additional file 6: Figure S3B).

For the other 8 solid cancers that were analysed by SomInaClust, between 3 (ovarian cancer) and 343 (colon cancer) putative driver genes were identified (Additional file 7: Figure S4 and Additional file 4: Table S3).

### SomInaClust accurately distinguishes oncogenes from tumour suppressor genes

Based on the Vogelstein’ method we assume that at least 20% of the mutations occurring in OGs are located in mutational hot spots (i.e. have an OG score equal or higher than 20), whereas in TSGs at least 20% of the mutations are protein-truncating mutations (i.e. have a TSG score equal or higher than 20) [1]. Using this criterion, all putative driver genes were classified based on their OG and TSG scores. The classification accuracy can be estimated for the genes that belong to the CGC gene list, assuming that OGs are dominant and TSGs recessive cancer genes [4]. Based on this assumption, 90% (19 out of 21) of the candidate breast cancer genes, for which information is available in the CGC list, were correctly classified. These genes included both well-known oncogenes such as PIK3CA, AKT1 and ERBB2 and well-known tumour suppressor genes such as TP53, PTEN and MAP3K1 (Additional file 4: Table S3). The only 2 genes that were not classified correctly were RUNX1 (a dominant gene classified as TSG) and DNMT3A (a recessive gene classified as OG).

**Figure 3:**
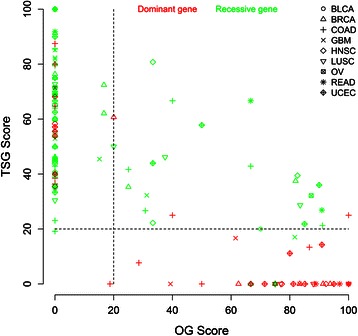
Classification accuracy of SomInaClust on the pooled set of putative driver genes in 9 solid cancers. TSG scores are plotted as a function of OG scores for all genes that were 1) retrieved by SomInaClust in the 9 solid cancers analysed and 2) present in the CGC list. Genes that are known to be dominant are shown in red, while recessive genes are shown in green. The legend indicates the cancer type and is shown on the top right. Dashed lines indicate cut-offs for classification as oncogene or as tumour suppressor genes. Genes in the lower right part are classified as oncogenes, in the upper (left and right) part as tumour suppressor genes, while genes in the lower left part are unclassified.

After pooling the results of all 9 cancers together, the overall classification accuracy was 81%. From the genes classified as OGs, 89% were predicted correct (34 out of 38). For the predicted TSGs this was 78% (91 out of 117) (Additional file 8: Table S4). This lower predictive value of TSG classified genes is for the major part explained by genes having high TSG scores and low OG score (Figure 3). Examples of these genes are NOTCH1, NOTCH2 and RUNX1 (Additional file 4: Table S3).

### SomInaClust is complementary to other candidate driver gene identification methods

**Figure 4:**
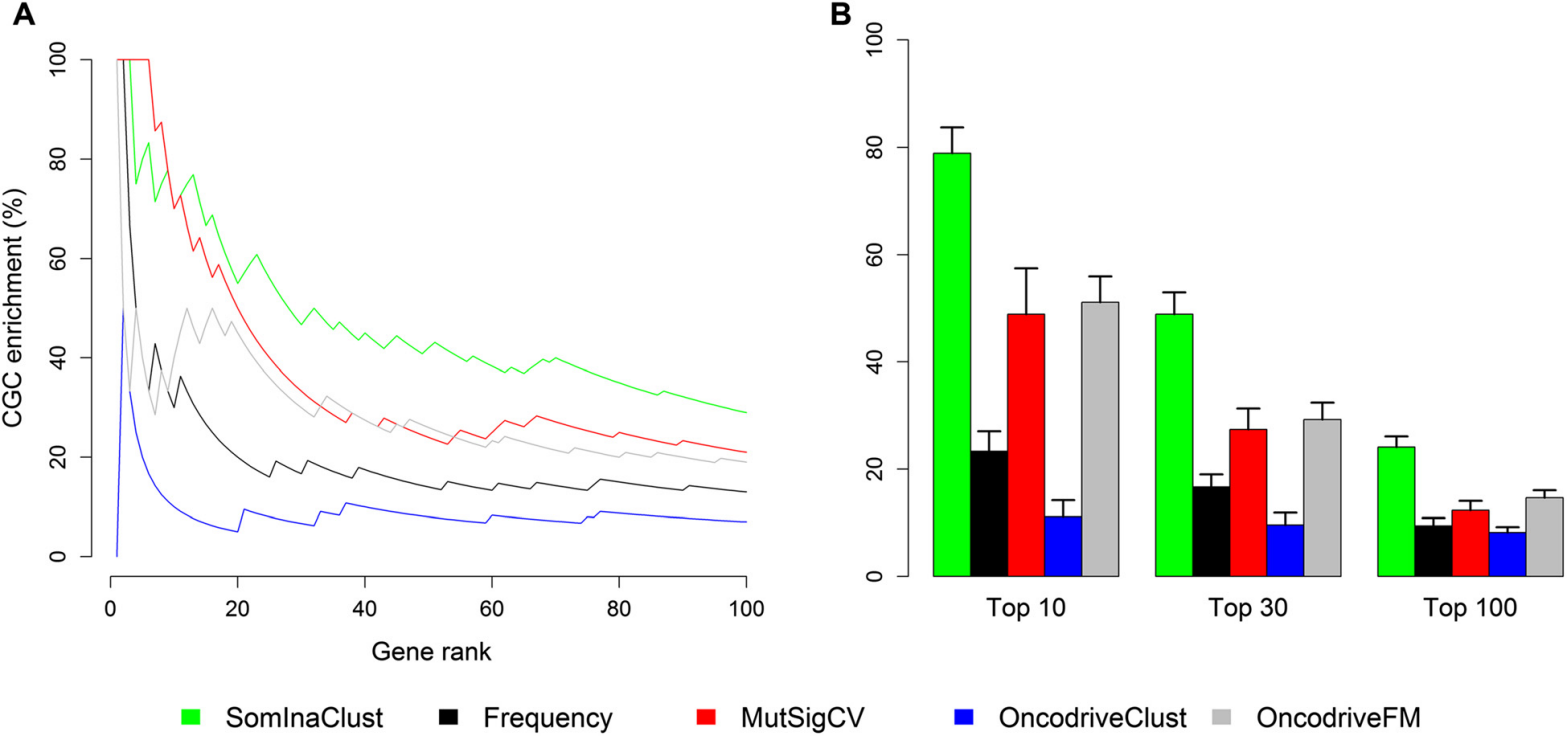
CGC enrichment of the genes identified in breast and other solid cancers. **(A)** Putative driver genes were detected on the TCGA breast cancer data set by different methods and ranked according to their increasing q values. For each 1–100 top ranked genes (x-axis) the proportion of CGC genes (y-axis) was determined. **(B)** The average CGC gene proportion of all 9 solid cancers that were analysed for the highest 10, 30 and 100 ranked genes by each method. Error bars indicate standard errors of the mean. The methods are indicated in the legend on the bottom of the figure.

Using the CGC list as a benchmark set for known cancer genes, it is possible to calculate the CGC enrichment, i.e. the proportion of the candidate driver genes that belong to the CGC list. When the putative driver genes retrieved with SomInaClust were ranked based on their (increasing) q values, a clear CGC gene enrichment was found for the top ranked genes for all 9 tumour types analysed (Figure 4A and Additional file 9: Figure S5). On average 78.9%, 48.9% and 24.1% of the top 10, 30 and 100 selected genes are known as cancer genes according to the CGC list (Figure 4B). A comparison of these CGC enrichment results with the results obtained by similar driver gene identification methods (i.e. MutSigCV, OncodriveClust and OncodriveFM), showed that the best CGC enrichments for a given number of ranked genes were obtained with SomInaClust (Figure 4B and Additional file 9: Figure S5). Furthermore, this enrichment also outperforms a simple mutation’s frequency-based enrichment, sustaining the relevance of the prioritization and deprioritization of that was shown in Additional file 6: Figure S3.

**Figure 5:**
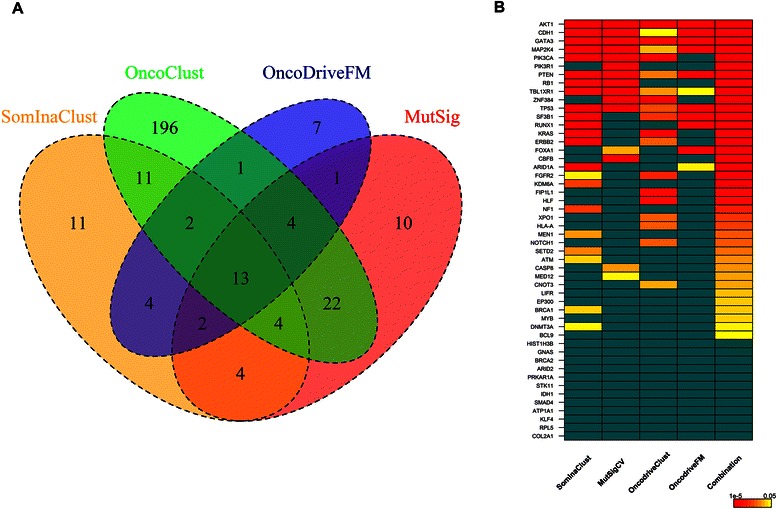
Putative breast cancer genes retrieved by 4 different methods. **(A)** Venn diagrams indicating the total number of putative breast cancer driver genes retrieved by 4 different methods (method names are indicated on the diagram). **(B)** Comparison of the significance levels of the genes that were detected by the 4 methods. Genes are ranked according to the product of the q values of the 4 methods together (indicated by the last “combination” column). The colour scale is shown on the bottom right with q values varying from 0.05 (yellow) to 1^e^-5 or higher (red). Blue boxes indicate non-significant genes.

Compared to the 51 genes retrieved by SomInaClust, the 3 alternative methods we compared our results with, retrieved respectively 60 (MutSigCV), 253 (OncodriveClust) and 34 (OncodriveFM) putative driver genes on the breast cancer dataset (Figure 5A). From these gene sets, thirteen genes were retrieved by all 4 methods (Figure 5A). These genes included not only well known cancer genes (e.g. TP53, PTEN, AKT1) but also genes with a less clear (breast) cancer association (e.g. RBMX, RBM5). All 4 methods were found to be highly complementary as several genes were only retrieved by a single method i.e. 11 genes were detected by SomInaClust only, 10 by MutSigCV, 196 by OncodriveClust and 7 by OncodriveFM (Figure 5A). From these gene sets, 7 (64%), 4 (40%), 6 (3%) and 0 genes correspond to known cancer genes respectively, according to the CGC gene list (Figure 5B). The 7 CGC genes that were detected by SomInaClust only included well-known (germline) cancer genes such as BRCA1, MEN1 and NF1. The complementarity of the 4 methods is further supported by the fact that some known cancer genes (e.g. EP300, LIFR and BCL9) were not detected by any individual method, but could be detected by the combination of all methods (Figure 5B). The results of the comparison of the putative driver genes retrieved in the 8 other solid cancers are shown in Additional file 10: Figure S6.

### The identified driver genes are active in cancer pathways

**Figure 6:**
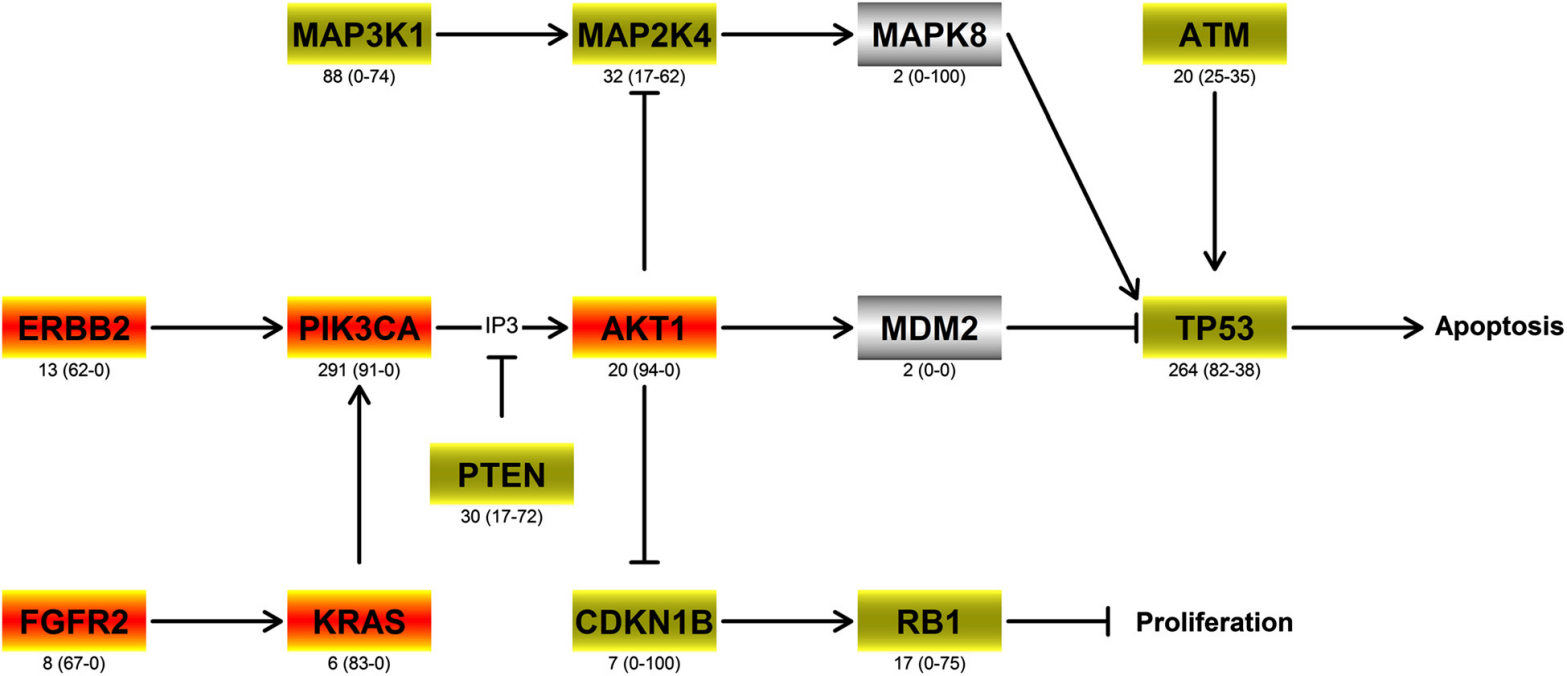
Signalling network of the putative breast cancer genes. The top-ranked genes that were detected on the breast cancer dataset were shown to be primary involved in the PI3K and the MAPK pathways. Genes are coloured green when they were classified as putative tumour suppressor genes (TSG scores ≥20) or in red when classified as putative oncogenes (OG scores ≥20 and TSG scores <20). The genes indicated in grey were not found to be significant by SomInaClust. Mutation frequencies and scores (OG score and TSG score respectively) are indicated below each gene. Arrows indicate stimulation, while bar-headed lines indicate inhibition.

To verify whether the retrieved genes are active in known cancer pathways, a KEGG pathway enrichment analysis was performed. This analysis showed that the identified putative breast cancer driver genes are indeed active in pathways involved in apoptosis, PI3K-AKT, MAPK and ERBB signalling (Additional file 11: Table S5). Figure 6 summarizes the interaction between the identified genes in these pathways. For all of these genes, their role in carcinogenesis (i.e. the evolution towards a selective growth advantage) can be directly derived from the SomInaClust classification as a putative OG or TSG, with the former leading to pro-proliferative or anti-apoptotic effects and the latter to the opposite effects. Interestingly, although some rarely mutated genes like KRAS, ERBB2 and CDKN1B were identified by SomInaClust and have obvious carcinogenic roles in the aforementioned pathways, also genes that remained below the detection threshold (i.e. qDG > 0.05) had OG and TSG scores showing a clear tendency towards a correct classification, based on their signalling interactions (e.g. MAPK8).

Similar KEGG driver pathways were found for the other 8 tumours that were analysed using SomInaClust (Additional file 11: Table S5) and for most of the genes that are active in these signalling pathways, their predicted role (OG or TSG) is related to carcinogenesis.

## Discussion

In this study we have introduced SomInaClust, a new cancer (driver) gene identification method that is based on the observation that somatic driver mutations in cancer tend to be inactivating (protein-truncating) and/or cluster together across tumour samples. This mutation pattern is further used to classify putative cancer genes in OGs or TSGs. The method is based on a principle described by Vogelstein et al. [1] and prioritizes driver genes by calculating a driver gene statistic (i.e. qDG-value).

SomInaClust uses prior information regarding mutational hot spots and the background mutation rate. In this study this prior information was determined from COSMIC, but any other large mutation database could have been used for this purpose. Using such a large reference database allows identifying hot spots that are impossible to recover at the specific cancer tissue type level due to the small size of these specific datasets (i.e. it is impossible to detect mutation clusters when only a few mutations are present). As an alternative, more sophisticated methods could have been used for the detection of these mutation clusters (e.g. iPAC and GraphPac [24,25]), but, as these depend on the availability of additional knowledge (e.g. regarding protein spatial structure information), we chose for the more widely applicable statistical approach presented in this work. This approach was shown to identify mutation clusters in 867 genes, including most well-known oncogenic mutations (e.g. in oncogenes like EGFR, PIK3CA and AKT1).

SomInaClust was shown to identify candidate driver genes with high accuracy. Even genes that are mutated in less than 1% of all sequenced tumour samples were detected. This is exemplified by the analysis of the breast cancer dataset, from which the well-known but rarely mutated cancer genes CDKN1B (7 mutations out of 772 samples), KRAS (6 mutations) and MEN1 (5 mutations) were identified. On the other hand, the method was also shown to deprioritize frequently mutated genes like TTN, MUC4 and RYR2, which have been described as “artefacts” by others [10,26].

We compared the results obtained with SomInaClust with those obtained by the previously published driver gene prioritization methods MutSigCV, OncodriveFM and OncodriveClust [11,14,15] on the same dataset. Genes identified by SomInaClust as well as by the other investigated methods not only included well-known cancer genes, but also genes without a previously described cancer link (e.g. RBMX and TBM5 on the breast cancer dataset). As every individual method uses a partially different approach (different background mutation models, mutation clustering, functional impact) to prioritize drivers, it is very likely that genes identified by multiple methods are truly involved in (subgroups of) breast carcinogenesis. The analysis in this study also confirmed the known complementarity of the different driver gene prioritization methods [12,27]. Indeed, every single method also identified unique gene sets (i.e. genes identified by one single method). Although some of these genes probably represent false positive results, it is interesting to note that SomInaClust contains amongst its predicted driver genes, the highest proportion (64%) of previously described cancer (CGC) genes. In line with these findings, the SomInaClust driver gene prioritization, as quantified via a CGC enrichment analysis, was found to be superior to the other methods that were examined.

Of all the investigated methods, SomInaClust was the only method to identify 10 genes (e.g. BRCA1, ATM, NF1) based on their higher than expected number of protein-truncating mutations. This higher sensitivity towards the detection of these well-known tumour suppressor genes is likely explained by the fact that SomInaClust takes into account all protein-truncating mutations, rather than just searching for clustering mutations, which is done by e.g. OncodriveClust [14].

Apart from the methods mentioned here, several other driver gene prioritization methods have been published (for a review see [12]). However, while the methods that were evaluated in this study only require a mutation file to be run, most of the other available methods require additional data (e.g. bam files for MuSiC [13]).

Under the assumption that OGs become carcinogenic by dominant mutations (i.e. by mutations in either allelic variants of a gene), and TSGs by recessive mutations (i.e. both allelic variants of a gene need to be mutated) [4], the classification accuracy could be calculated for the putative driver genes that were present in the CGC list. This resulted in an overall classification accuracy of 81% (positive predictive values of 89% for OGs and 78% of TSGs) for the 9 tumours that were analysed in this study. The lower predictive values for TSGs may be an underestimation. Firstly, because it has been shown that some genes have dual roles in cancer, in which they function as an OG in one cancer type but as a TSG in another. This is exemplified by NOTCH1, which has been described as a dominant (and hence OG) gene in leukaemia, but was shown to be a TSG in solid cancers as well [28]. Also in this study, NOTCH1 was (wrongly?) classified as a TSG in head and neck cancer, possibly explaining part of the misclassified genes and indicating that the true classification accuracy is likely to be higher. Secondly it has been demonstrated that the assumption that TSGs act in a recessive way does not always hold. Indeed, haploinsufficiency has been shown for several TSGs, i.e. genes that result in a growth advantage even when only one allele is defective [29]. Interestingly, this has also been shown for RUNX1, one of the only 2 putative driver genes that were misclassified in breast cancer [30].

The highest number of putative driver genes was found in colon (343) and uterine cancer (308), while in ovarian cancer only few genes were retrieved. This may be related to the recently described pan-cancer classes, with the first 2 cancers belonging to the M class (dominated by mutations) and the last one belonging to the C class (dominated by copy number variations) [31]. Interestingly, a lot of rarely mutated putative driver genes that were detected in breast cancer have been described as copy number amplifications (e.g. ERBB2) or deletions (e.g. RB1, MEN1, PTEN) before [9]. The identification of these genes as being mutated at the individual patient level might be crucial for personalized treatment choices.

## Conclusions

In this study a method was presented that was shown to be able to detect candidate cancer driver genes based on somatic mutation patterns of inactivating and clustering (SomInaClust). The method was shown to be complementary to existing similar-purpose methods with the additional advantages that it has a higher sensitivity, also for very rare (<1%) mutations, and it accurately classifies the detected genes into putative oncogenes and tumour suppressor genes.

The method, example files, reference files and information on how to use the method are implemented as an R package, which is freely available at http://bioinformatics.intec.ugent.be/sominaclust.

## Additional files

Additional file 1: Figure S1. Outline of the SomInaClust method. For each gene the total number of mutations is counted across all tumour samples in a reference database (step 1: reference step) or a test dataset (step 2: gene detection step). Protein truncating mutations (i.e. nonsense mutations or out-of-frame indels) are referred to as TSG mutations. Other mutations (i.e. missense mutations or in-frame indels are referred to as OG mutations). N: the (uncorrected) number of silent (N_sil_), OG (N_OG_) and TSG (N_TSG_) mutations. n: the number of OG (n_OG_), TSG (n_TSG_) and total number (n_tot_) of mutations, multiplied with the gene-specific OG/TSG correction factor. k: number of OG mutations located at the same CDS position (k_OG_) or at mutational hot spot locations (k_hOG_), multiplied with the gene-specific OG correction factor. See main text (Methods section) for further explanation.
Additional file 2: Table S1. Overview of all theoretically possible mutations occurring in non-stop codons. Each of 61 non-stop codons can theoretically mutate in 9 different codons (3 possible mutations for each nucleotide), implying a total number of 549 possible mutations. From these 549 mutations, 134 can be classified as silent, 392 as missense and 23 as nonsense. Mutation types are indicated with 0 (absent) or 1 (present). nt1, nt2 and nt3: first, second and third nucleotide of the corresponding codon.
Additional file 3: Table S2. Overview of genes and their identified mutational hot spots in the COSMIC v71 database. The first tab shows the genes that contain at least 1 identified mutational hot spot. Genes are ranked according to their number of detected hot spots. Columns represent gene names, number of detected hot spots, size of the smallest mutation cluster and number of mutations respectively. The second tab shows the CDS and amino acid positions of all the identified mutational hot spots.
Additional file 4: Table S3. Putative driver genes detected by SomInaClust in 9 different cancer types. Genes are ranked according to their increasing q values. Columns represent gene names, number of mutations, q value, OG score, TSG score, classification (OG or TSG) and CGC class (dominant or recessive, NA means the gene does not belong to the CGC list). The following cancer types were analysed (tab names): bladder cancer (BLCA), breast cancer (BRCA), colon cancer (COAD), glioblastoma multiforme (GBM), head and neck squamous cell cancer (HNSC), lung squamous cell cancer (LUSC), ovarian cancer (OV), rectal cancer (READ) and uterine cancer (UCEC).
Additional file 5: Figure S2. Complementary role of clustering and protein-truncating mutations in the detection of putative cancer genes. Comparison of the significance levels of the genes that were detected by the SomInaClust method between using qDG (default), using only the clustering mutations (i.e. OG mutations, yielding qOG) and using only the protein-truncating mutations (i.e. TSG mutations, yielding qTSG). Genes are ranked on increasing qDG values and only the first 50 genes are shown. The colour scale is shown on the bottom right with q values varying from 0.05 (yellow) to 1^e^-5 or higher (red). Blue boxes indicate non-significant genes. The following cancer types were analysed as indicated on top of each figure: bladder cancer (BLCA), breast cancer (BRCA), colon cancer (COAD), glioblastoma multiforme (GBM), head and neck squamous cell cancer (HNSC), lung squamous cell cancer (LUSC), ovarian cancer (OV), rectal cancer (READ) and uterine cancer (UCEC).
Additional file 6: Figure S3. Prioritization and deprioritization of putative cancer genes by SomInaClust. Correlation between SomInaClust (y-axis) and mutation frequency (x-axis) ranks in breast cancer. (A) Focus on the first 20 SomInaClust ranked genes. (B) Focus on the first 20 frequency ranked genes. Genes that are clearly (de)prioritized are indicated and named in red.
Additional file 7: Figure S4. Putative driver genes retrieved in 8 solid cancers. Pyramid plots showing the top (20) putative driver genes retrieved by SomInaClust in 8 TCGA cancers: bladder cancer (BLCA), colon cancer (COAD), glioblastoma multiforme (GBM), head and neck squamous cell cancer (HNSC), lung squamous cell cancer (LUSC), ovarian cancer (OV), rectal cancer (READ) and uterine cancer (UCEC). Genes are ranked according to increasing q values. The 20 highest ranked genes are shown, or less when fewer genes were found significant. The OG scores are visualized on the left and the TSG scores on the right. Dotted lines indicate cut-offs for classification as oncogene or tumour suppressor gene.
Additional file 8: Table S4. Classification results. Contingency tables showing the classification results of all 9 individual cancers and the pooled set of tumours. The following cancer types were analysed: bladder cancer (BLCA), breast cancer (BRCA), colon cancer (COAD), glioblastoma multiforme (GBM), head and neck squamous cell cancer (HNSC), lung squamous cell cancer (LUSC), ovarian cancer (OV), rectal cancer (READ) and uterine cancer (UCEC).
Additional file 9: Figure S5. CGC enrichment of the top 100 ranked genes in 8 solid cancers. CGC enrichment plots for 8 different TCGA cancers: bladder cancer (BLCA), colon cancer (COAD), glioblastoma multiforme (GBM), head and neck squamous cell cancer (HNSC), lung squamous cell cancer (LUSC), ovarian cancer (OV), rectal cancer (READ) and uterine cancer (UCEC). Genes are ranked according to increasing q values and for each top 1–100 ranked genes (x-axis) the proportion of CGC genes (y-axis) is determined. Five different methods were compared as indicated in the legend on the top right of each plot.
Additional file 10: Figure S6. Putative driver genes retrieved by 4 different methods in 8 solid cancers. (A) Venn diagrams indicating the total number of putative cancer driver genes retrieved by 4 different methods. (B) Comparison of the significance levels of the genes that were detected by the 4 methods. Genes are ranked according to the product of the q values of the 4 methods together (indicated by the last “Combination” column). The colour scale is shown on the bottom right with q values varying from 0.05 (yellow) to 1^e^-5 or higher (red). Blue boxes indicate non-significant genes. The following cancer types were analysed as indicated on top of each figure: bladder cancer (BLCA), colon cancer (COAD), glioblastoma multiforme (GBM), head and neck squamous cell cancer (HNSC), lung squamous cell cancer (LUSC), ovarian cancer (OV), rectal cancer (READ) and uterine cancer (UCEC).
Additional file 11: Table S5. KEGG pathway enrichment analysis of the putative driver genes. Pathways are ranked according to increasing q values. Columns represent cancer type, pathway IDs, pathway names and the enrichment q values respectively. The following cancer types were analysed (tab names): bladder cancer (BLCA), breast cancer (BRCA), colon cancer (COAD), glioblastoma multiforme (GBM), head and neck squamous cell cancer (HNSC), lung squamous cell cancer (LUSC), ovarian cancer (OV), rectal cancer (READ) and uterine cancer (UCEC).
