# Supplementary figures and images for: SomInaClust: detection of cancer genes based on somatic mutation patterns of inactivation and clustering
Source: BMC Bioinformatics. 2015 Apr 23;16:125. doi: 10.1186/s12859-015-0555-7 (PMC4410004; doi:10.1186/s12859-015-0555-7)

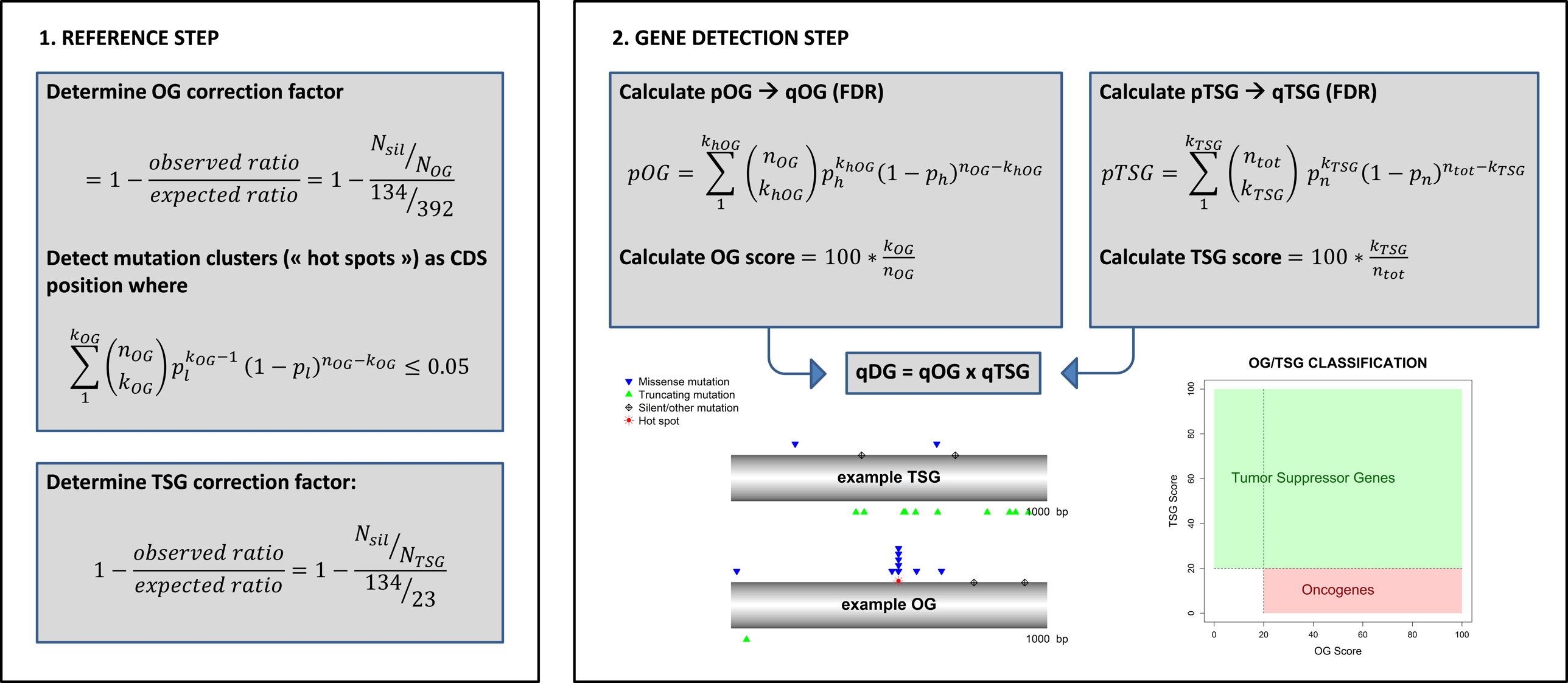

Supplement: Additional file 1: Figure S1. — Outline of the SomInaClust method. For each gene the total number of mutations is counted across all tumour samples in a reference database (step 1: reference step) or a test dataset (step 2: gene detection step). Protein truncating mutations (i.e. nonsense mutations or out-of-frame indels) are referred to as TSG mutations. Other mutations (i.e. missense mutations or in-frame indels are referred to as OG mutations). N: the (uncorrected) number of silent (Nsil), OG (NOG) and TSG (NTSG) mutations. n: the number of OG (nOG), TSG (nTSG) and total number (ntot) of mutations, multiplied with the gene-specific OG/TSG correction factor. k: number of OG mutations located at the same CDS position (kOG) or at mutational hot spot locations (khOG), multiplied with the gene-specific OG correction factor. See main text (Methods section) for further explanation. [file 12859_2015_555_MOESM1_ESM.png]

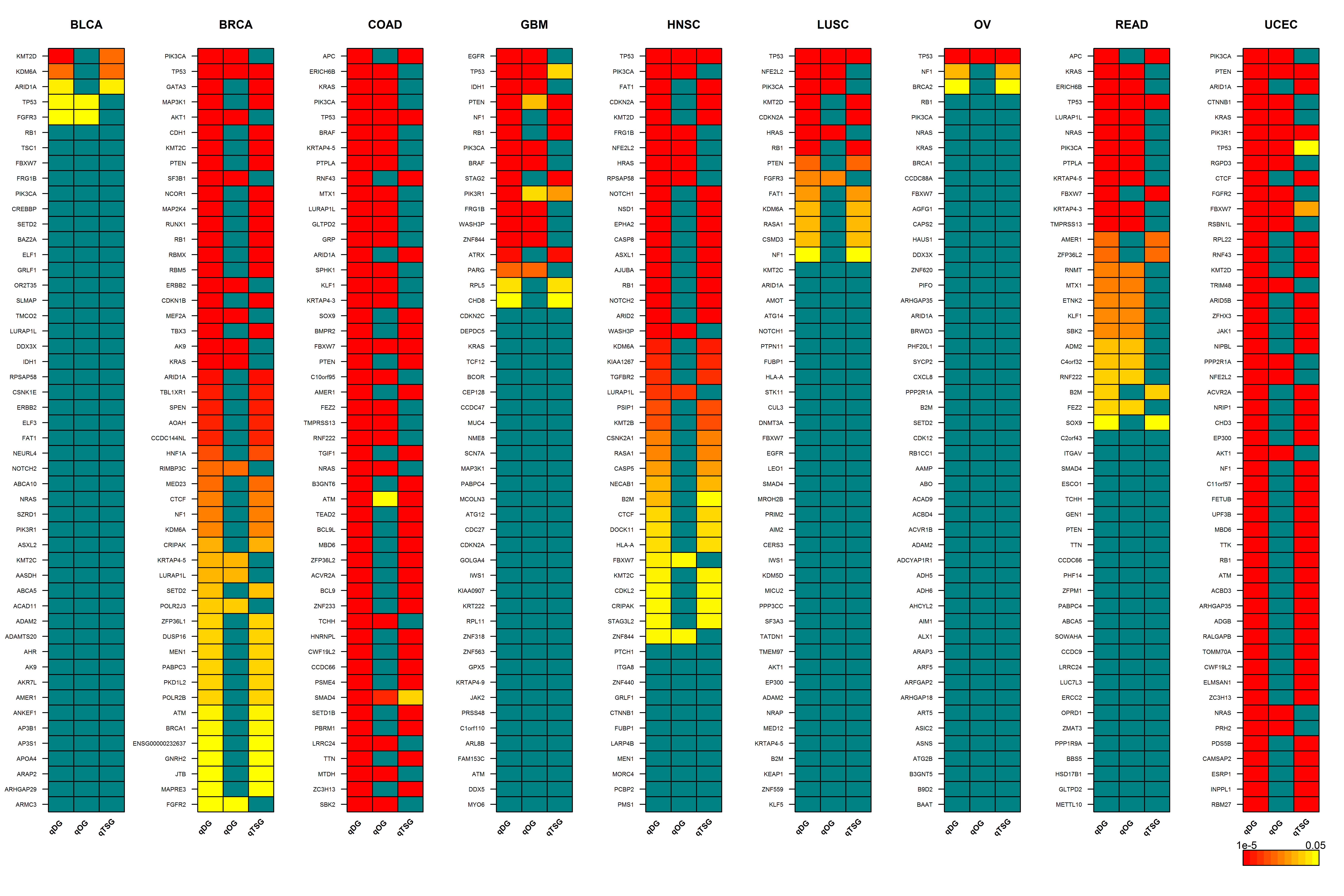

Supplement: Additional file 5: Figure S2. — Complementary role of clustering and protein-truncating mutations in the detection of putative cancer genes. Comparison of the significance levels of the genes that were detected by the SomInaClust method between using qDG (default), using only the clustering mutations (i.e. OG mutations, yielding qOG) and using only the protein-truncating mutations (i.e. TSG mutations, yielding qTSG). Genes are ranked on increasing qDG values and only the first 50 genes are shown. The colour scale is shown on the bottom right with q values varying from 0.05 (yellow) to 1e-5 or higher (red). Blue boxes indicate non-significant genes. The following cancer types were analysed as indicated on top of each figure: bladder cancer (BLCA), breast cancer (BRCA), colon cancer (COAD), glioblastoma multiforme (GBM), head and neck squamous cell cancer (HNSC), lung squamous cell cancer (LUSC), ovarian cancer (OV), rectal cancer (READ) and uterine cancer (UCEC). [file 12859_2015_555_MOESM5_ESM.png]

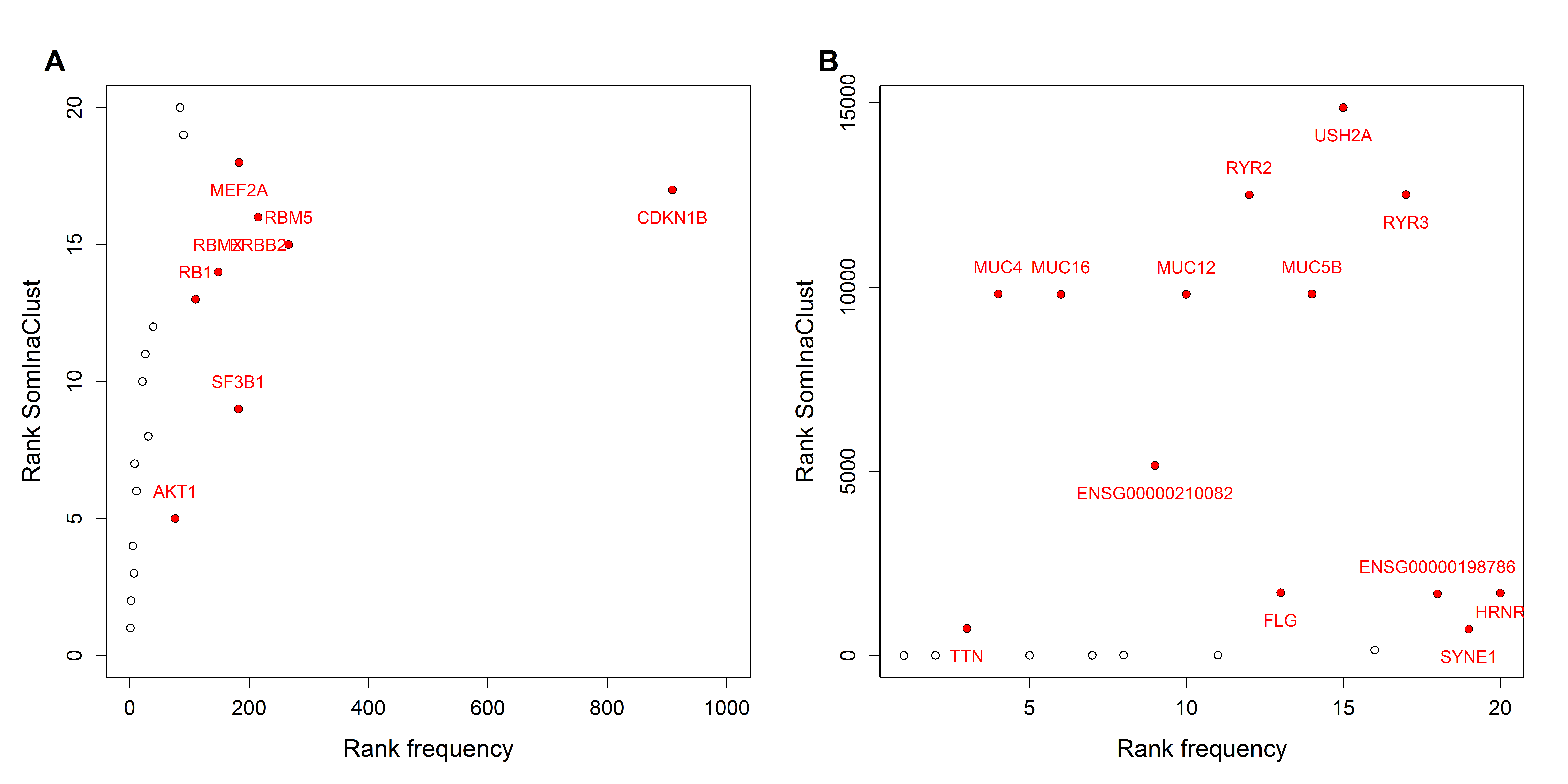

Supplement: Additional file 6: Figure S3. — Prioritization and deprioritization of putative cancer genes by SomInaClust. Correlation between SomInaClust (y-axis) and mutation frequency (x-axis) ranks in breast cancer. (A) Focus on the first 20 SomInaClust ranked genes. (B) Focus on the first 20 frequency ranked genes. Genes that are clearly (de)prioritized are indicated and named in red. [file 12859_2015_555_MOESM6_ESM.png]

BLCA

A

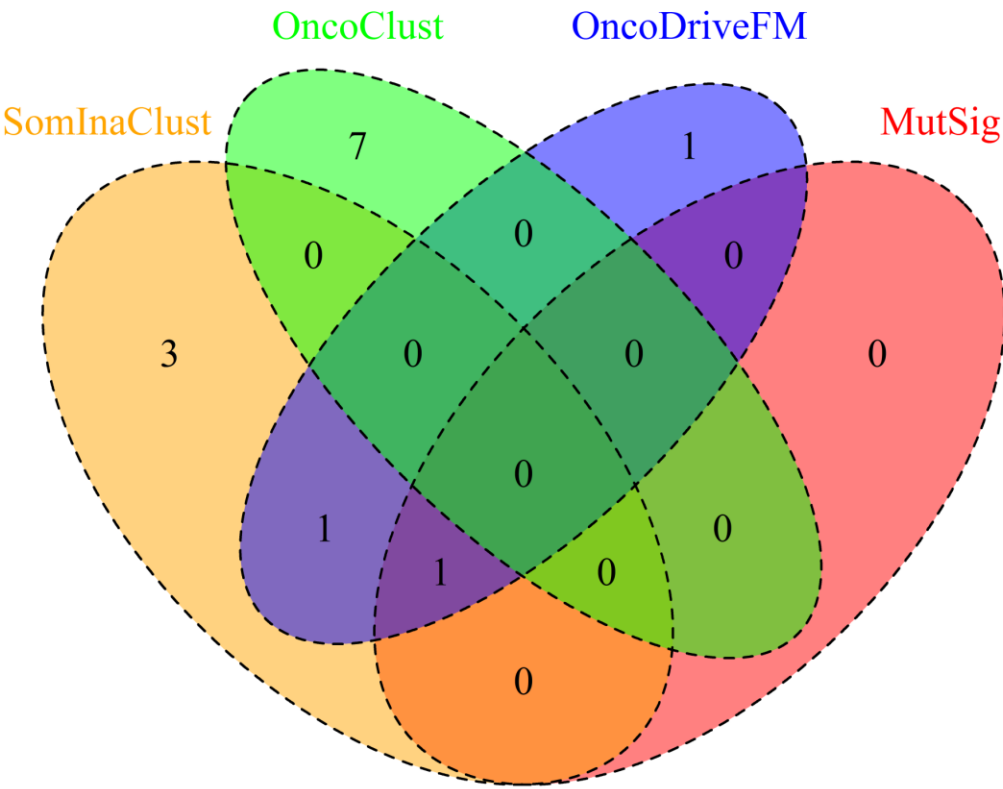

B

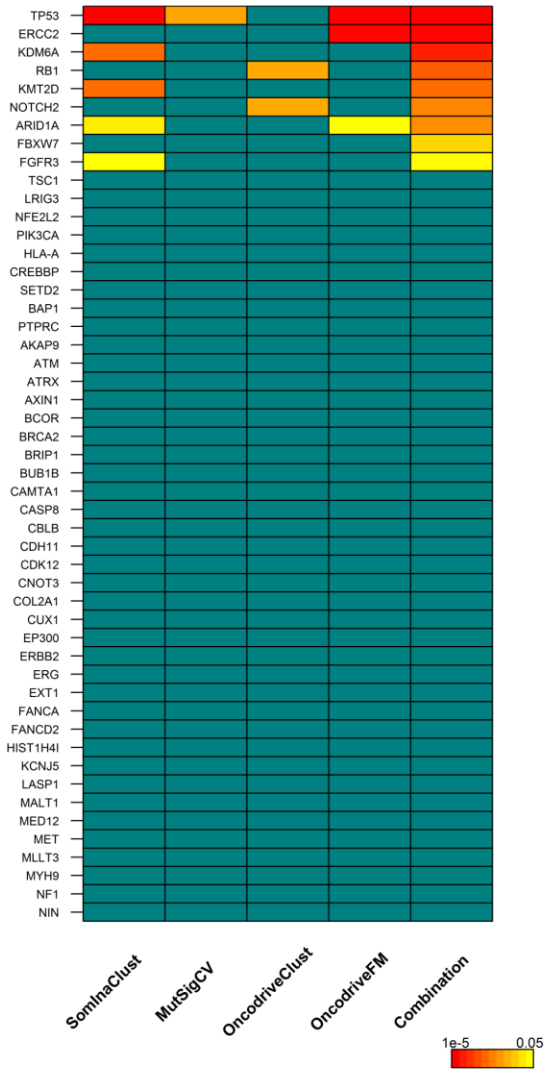

# COAD

A

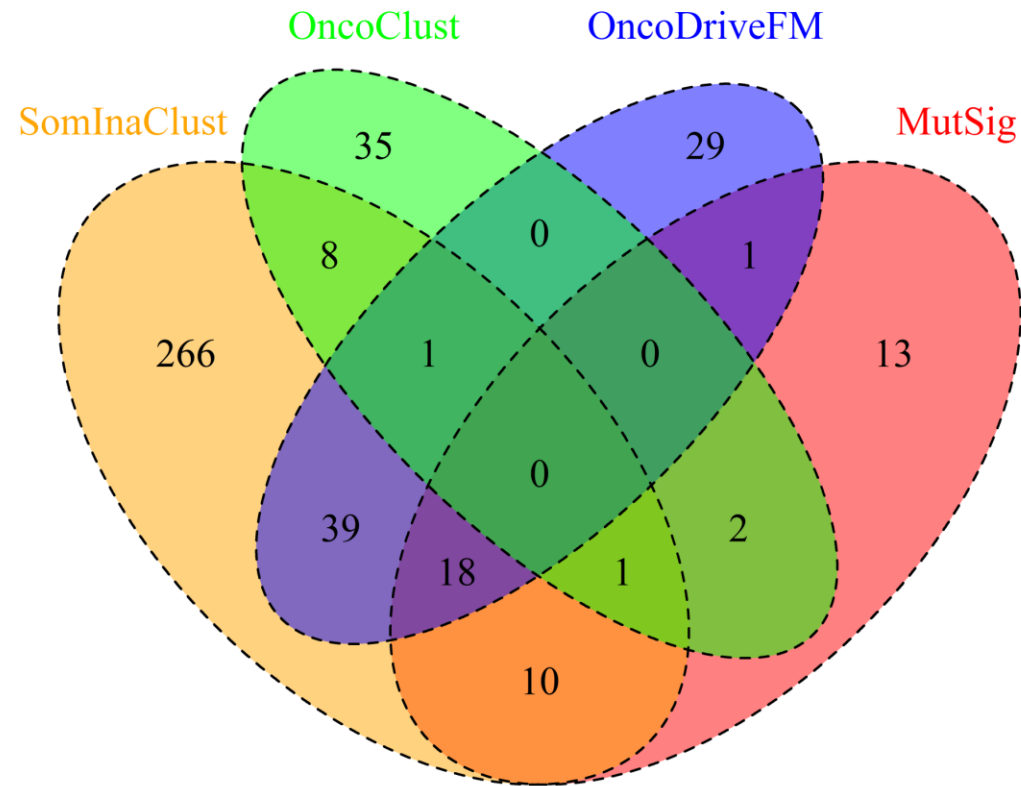

B

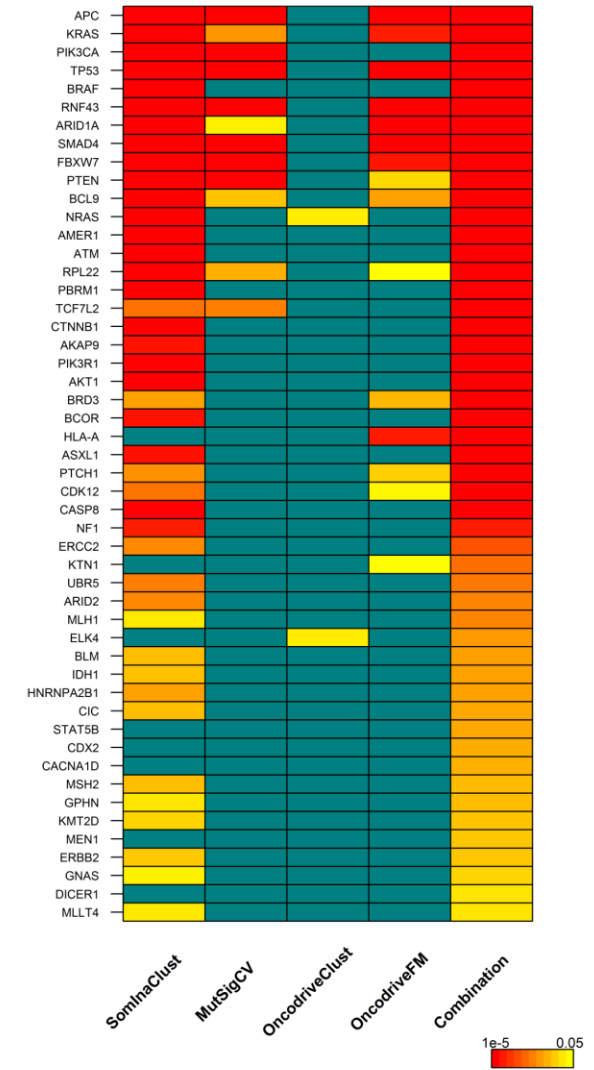

GBM

A

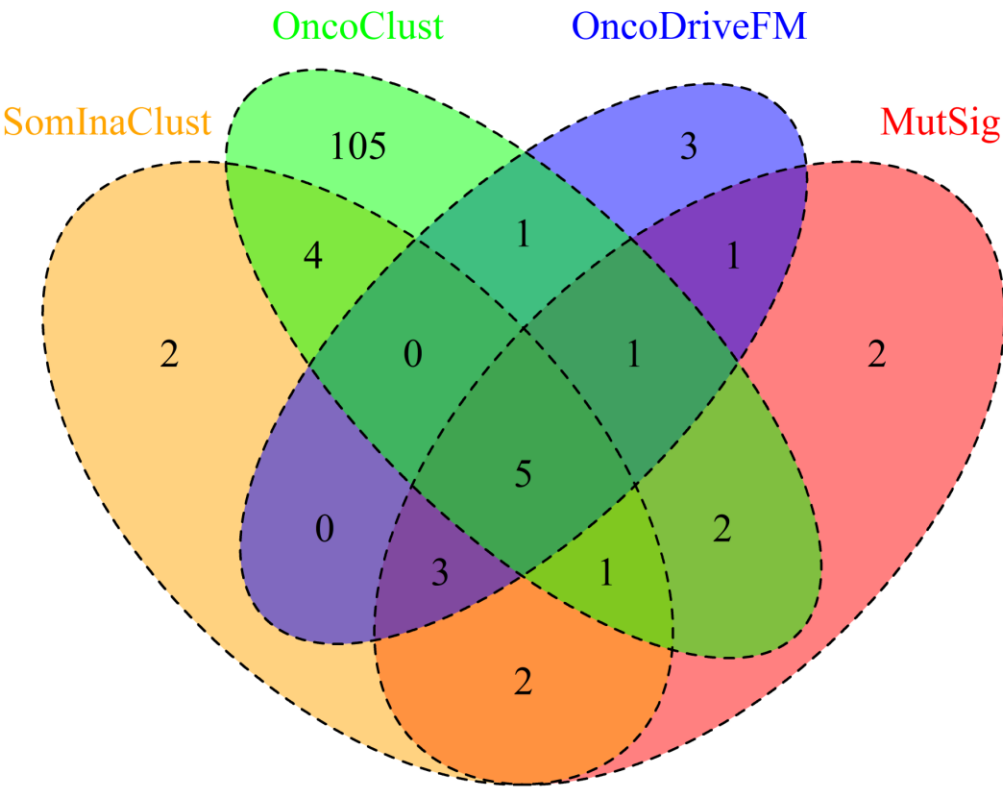

B

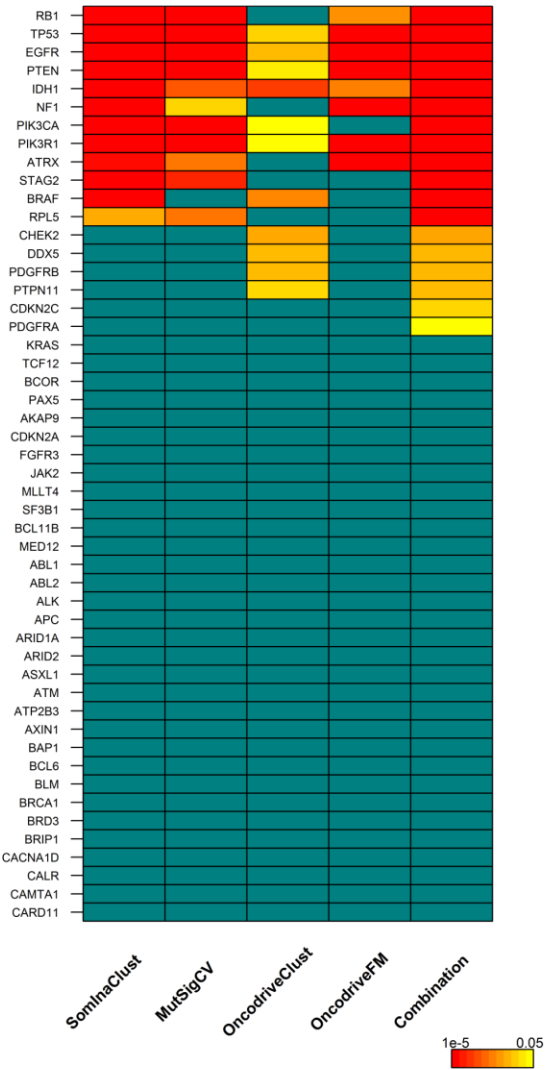

# HNSC

A

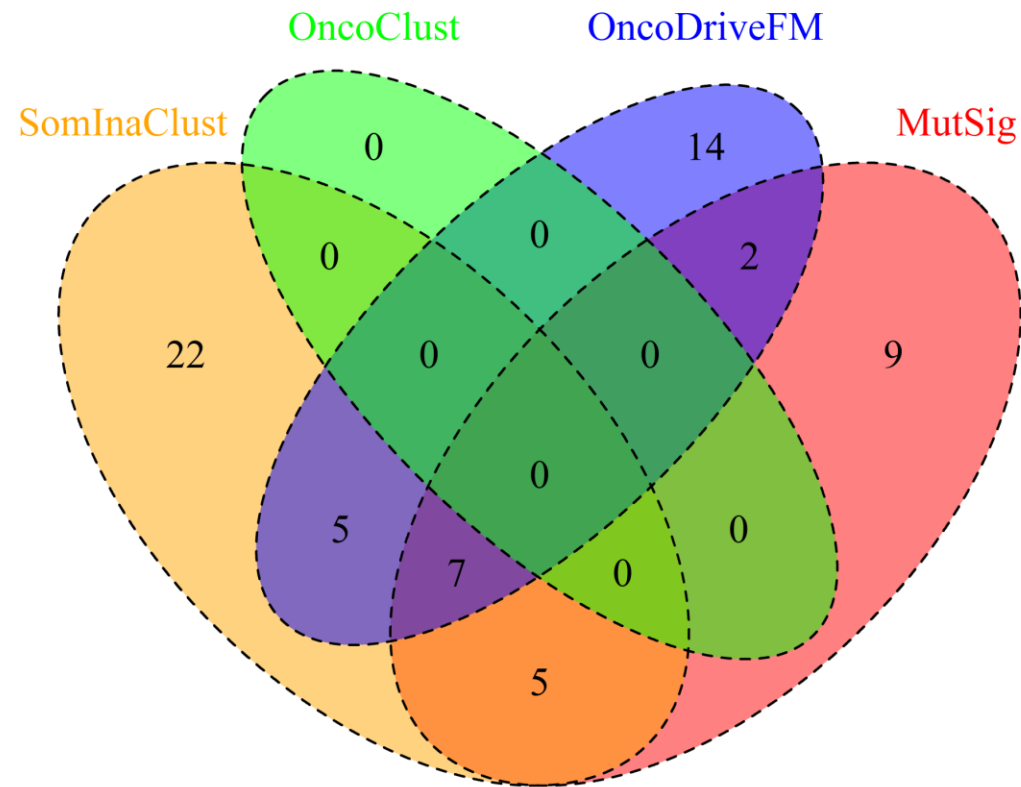

B

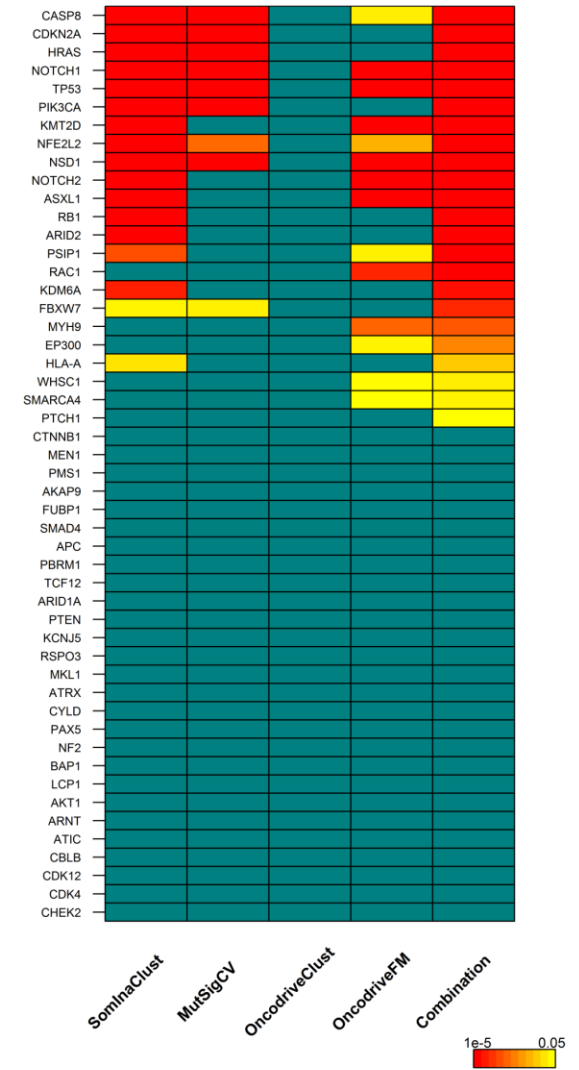

LUSC

A

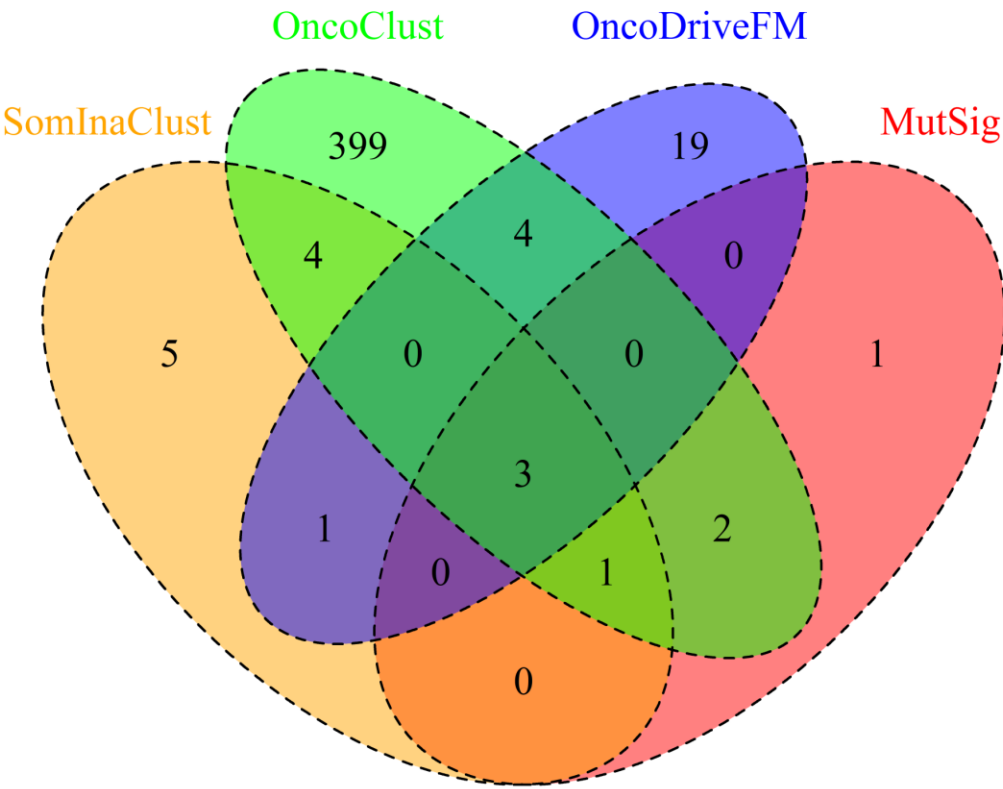

B

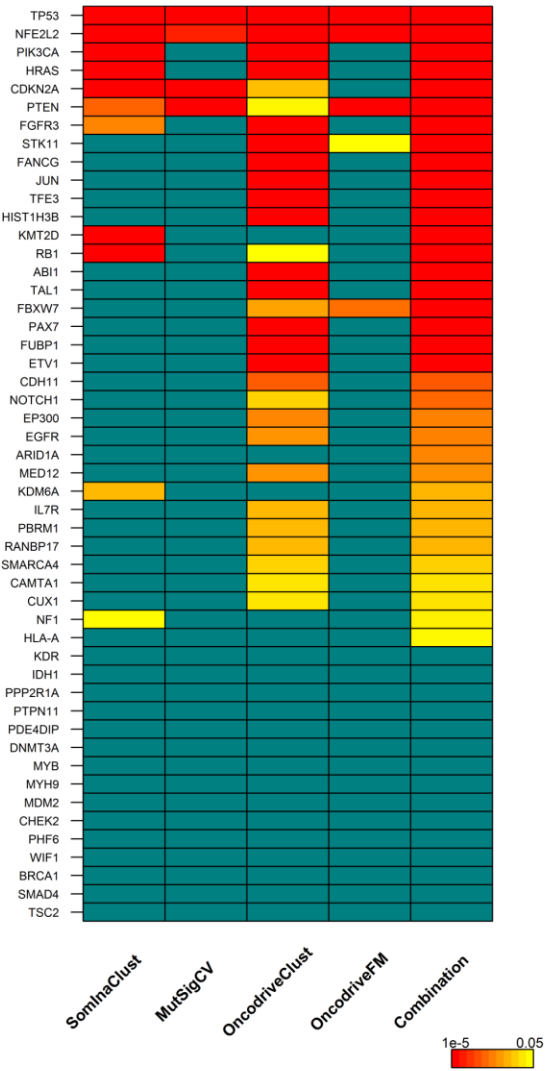

OV

A

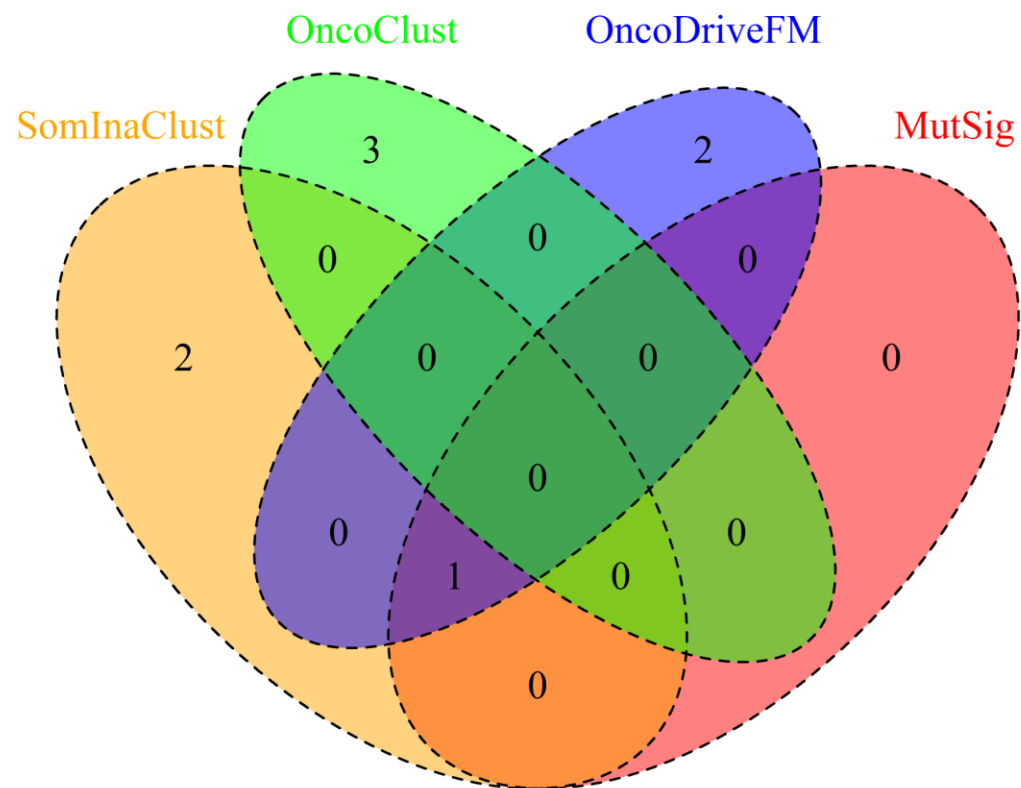

B

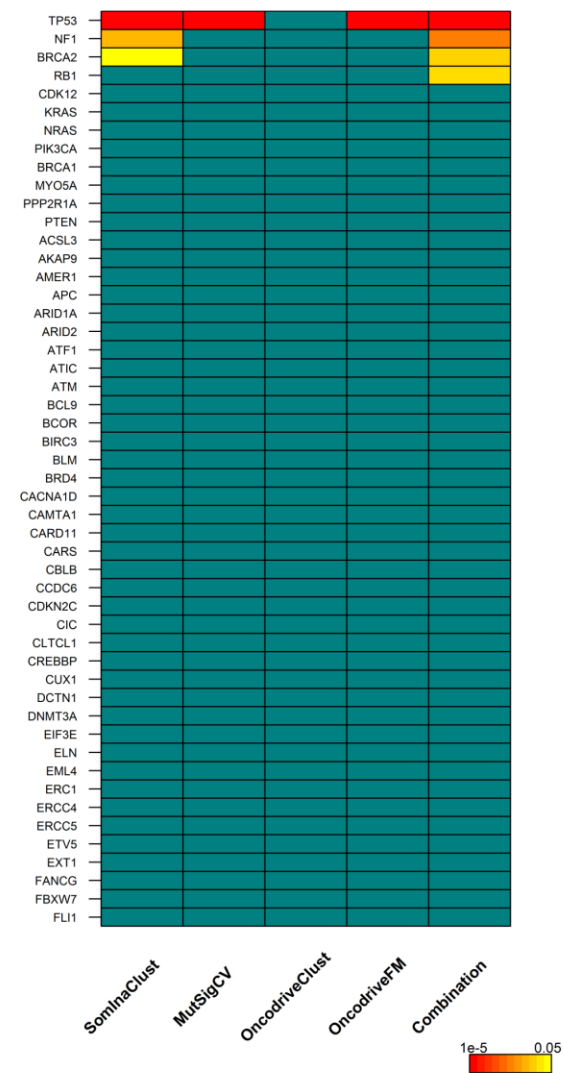

**B**

**A**

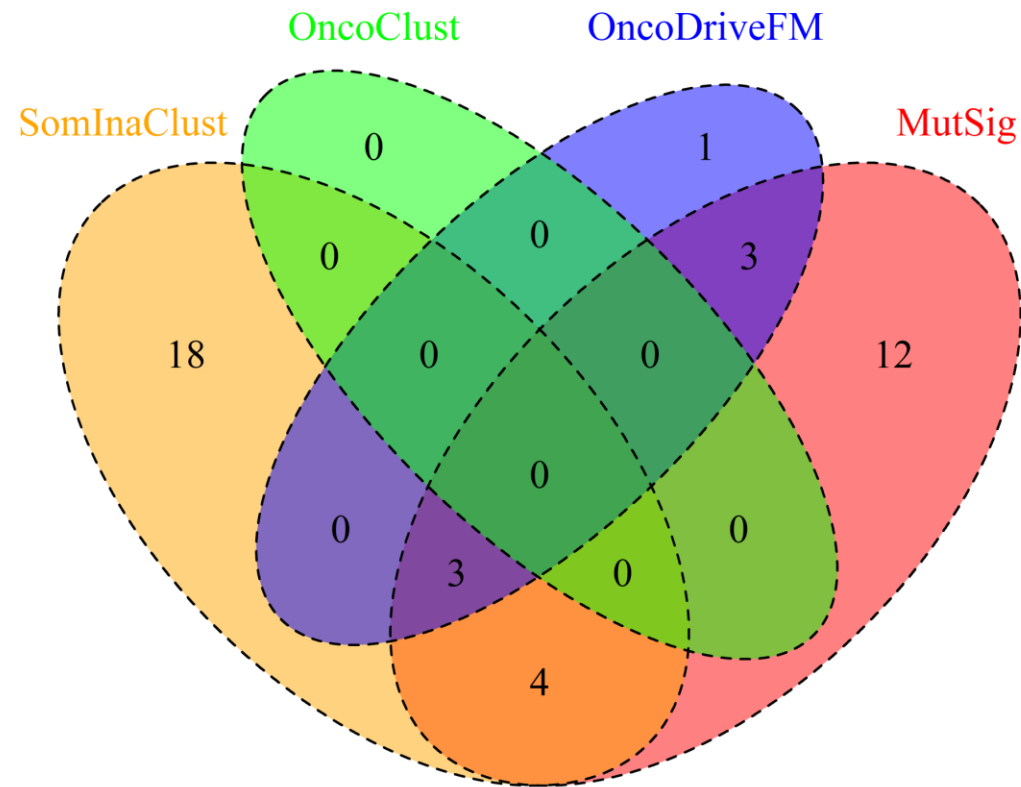

**B**

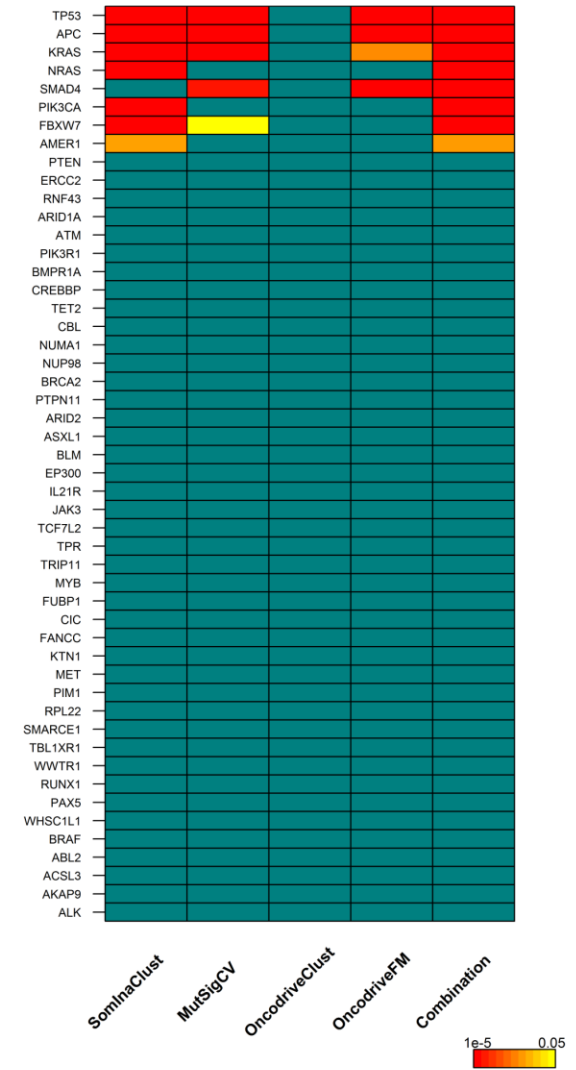

# UCEC

A

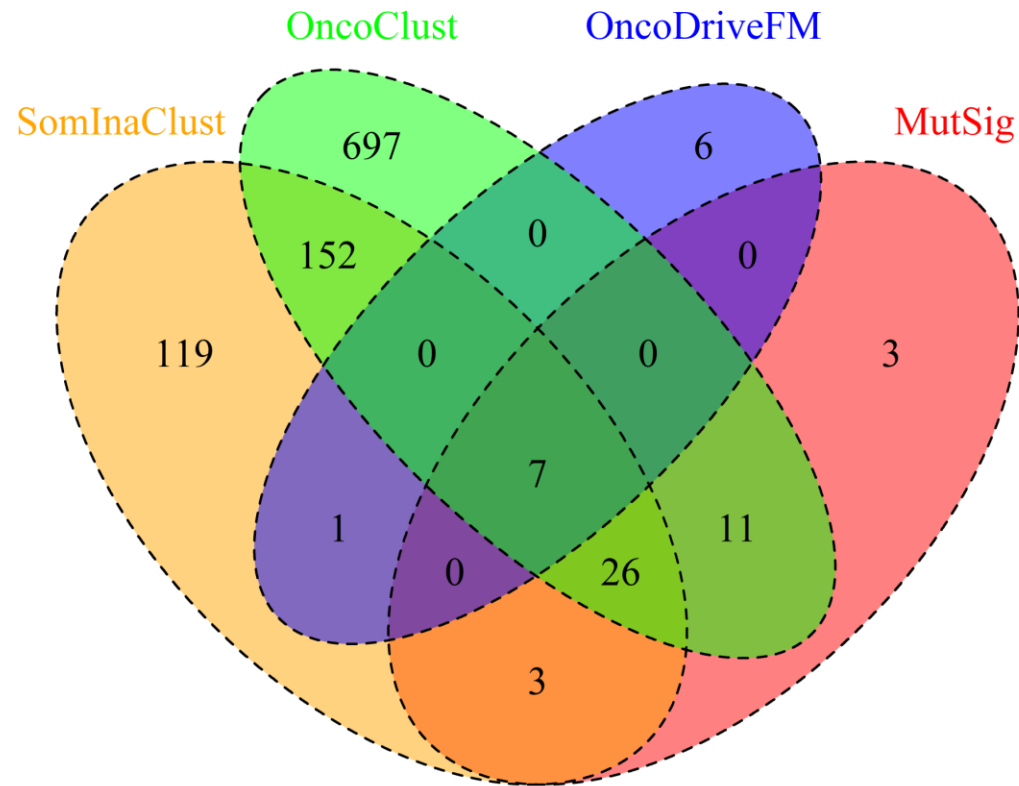

B

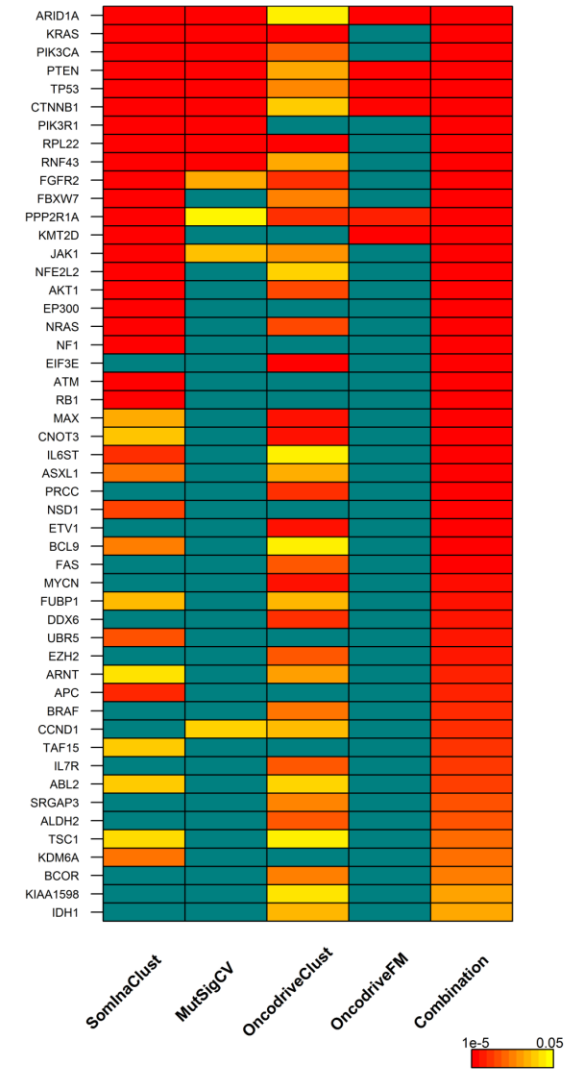

Supplement: Additional file 10: Figure S6. — Putative driver genes retrieved by 4 different methods in 8 solid cancers. (A) Venn diagrams indicating the total number of putative cancer driver genes retrieved by 4 different methods. (B) Comparison of the significance levels of the genes that were detected by the 4 methods. Genes are ranked according to the product of the q values of the 4 methods together (indicated by the last “Combination” column). The colour scale is shown on the bottom right with q values varying from 0.05 (yellow) to 1e-5 or higher (red). Blue boxes indicate non-significant genes. The following cancer types were analysed as indicated on top of each figure: bladder cancer (BLCA), colon cancer (COAD), glioblastoma multiforme (GBM), head and neck squamous cell cancer (HNSC), lung squamous cell cancer (LUSC), ovarian cancer (OV), rectal cancer (READ) and uterine cancer (UCEC). [file 12859_2015_555_MOESM10_ESM.pdf]
